# Effects of a purified krill oil phospholipid rich in long-chain omega-3 fatty acids on cardiovascular disease risk factors in non-human primates with naturally occurring diabetes type-2 and dyslipidemia

**DOI:** 10.1186/s12944-017-0411-z

**Published:** 2017-01-17

**Authors:** Petter-Arnt Hals, Xiaoli Wang, Yong-Fu Xiao

**Affiliations:** 1 Aker Biomarine Antarctic AS, Oksenoyveien 10, N-1366 Lysaker, Norway; 2 Crown Bioscience (Taicang) Inc., Science and Technology Park, 6 Beijing West Road, Taicang, Jiangsu Province People’s Republic of China

**Keywords:** Phospholipids, Omega-3 fatty acids, Choline, Cholesterol, Cardiovascular disease

## Abstract

**Background:**

High serum levels of cholesterol, in particular low-density lipoprotein cholesterol, are considered a significant risk factor for development of cardiovascular disease. Therefore, rigorous treatment regimens with statins and other pharmaceuticals have been used extensively to reduce elevated cholesterol levels. Literature data have not clearly concluded whether long-chain omega-3 fatty acids reduce, increase or leave circulating cholesterol unaffected. In the present study a novel krill-oil derived preparation of omega-3 rich phospholipids, mainly phosphatidylcholine, was administered orally at increasing doses for 12 weeks to dyslipidemic non-human primates, and cholesterols and several other risk factors for cardiovascular disease were measured before, during and after treatment.

**Methods:**

Six dyslipidemic non-human primates suffering from naturally occurring diabetes type-2 were included, three in a vehicle control group and three being treated with the omega-3 rich phospholipid preparation. The control and test items were given daily by gavage and the doses of the test item were 50, 150 and 450 mg phospholipids/kg/day. Each dose level was given for 4 weeks. Plasma concentrations of the omega-3 fatty acids were measured in connection with change in dose and the omega-3 index in erythrocytes was determined bi-weekly. Blood lipids, apolipoproteins and diabetes, inflammatory and safety biomarkers were determined either weekly, biweekly or every 4 weeks. For the blood lipids and apolipoproteins, control-adjusted mean values are presented while absolute values are presented for the other parameters. Due to the low number of animals in each group, no statistical analyses were done.

**Results:**

The only detectable effects measured during dosing with the lowest dose were an increase in HDL-cholesterol and apolipoprotein A1. The intermediate and high doses decreased total cholesterol, LDL-cholesterol, apolipoprotein B100 and triglycerides and increased HDL-cholesterol and apolipoprotein A1. No effects were seen on the diabetes and inflammatory markers and on safety biomarkers.

**Conclusions:**

The results indicate that the omega-3 rich phospholipid preparation had a positive impact on cardiovascular disease risk factors by reducing total cholesterol, LDL-cholesterol and triglycerides and increasing HDL-cholesterol. These findings justify further investigations of this preparation in animal models of dyslipidemia and, provided the current findings are confirmed, in human trials.

**Electronic supplementary material:**

The online version of this article (doi:10.1186/s12944-017-0411-z) contains supplementary material, which is available to authorized users.

## Background

Cholesterol is a natural component of mammalian cell membranes and is essential in this lipophilic barrier between cells. Lipoprotein complexes, which are composed of lipids and apolipoproteins, are required to transport cholesterol and lipids through the bloodstream. This transport system is crucial for life, but excessive concentrations of cholesterol in blood, especially when carried by low-density lipoproteins (LDLs) or very low density lipoproteins (VLDLs), can accumulate on the walls of arteries. These accumulated lipoproteins increase the risk for several cardiovascular diseases like stroke and ischemic heart disease including myocardial infarction, one leading cause of death in Western industrial societies. Hence, rigorous treatment regimens are often prescribed for patients diagnosed with elevated blood levels of cholesterols and in particular in the cases where LDL cholesterol (LDL-c) is above what is considered normal levels. For the last decades, inhibitors of the enzyme 3-hydroxy 3-methylglutaryl Coenzyme-A reductase (HMG CoA) have been the major treatment for the reduction of elevated cholesterol levels. These drugs, known as statins, inhibit HMG CoA and thereby one specific step in the cascade of cholesterol synthesis in cells and have proven clinically useful in the prevention of cardiovascular disease caused by hypercholesterolemia [1]. Recently, more novel drugs for reduction of circulating cholesterol, the proprotein convertase subtilisin/kexin type 9 (PCSK9) inhibitors, have been approved for clinical use. These new drugs act by reducing the endogenous production of PCSK9, which normally binds to the LDL receptor on hepatocytes and causes its internalization and lysosomal degradation, resulting in lower cellular concentrations of the LDL receptor and consequently a higher level of circulating LDL [2]. Other novel therapies for normalization of abnormal levels of circulating cholesterols include cholesteryl ester transfer protein (CETP) inhibitors like dalcetrapib [3], microsomal triglyceride transfer protein (MTP) inhibitors like lomitapide [4] and antisense therapy of which mipomersen is an FDA-approved drug [5].

Long-chain (LC) omega-3 fatty acids, in particular eicosapentaenoic acid (EPA; C20:5n3) and docosahexaenoic acid (DHA; C22:6n3), have long been assumed to have beneficial effects on cardiovascular health. Multiple biological effects known to be involved in the development of cardiovascular disease have been reported for these fatty acids, including reduction of blood triglycerides [6], improving endothelial function and reducing arterial wall stiffness [7, 8], reducing inflammations [9, 10], being antiarrhythmic [11], reducing blood pressure [12–14] and reducing overall risk for sudden cardiac death [15]. These two fatty acids are found only in marine sources like fat fish (e.g., salmon, mackerel and anchovies), krill and algae, and have been marketed for years as dietary supplements and more recently also as pharmaceutical products. The first approved pharmaceutical containing EPA and DHA was Omacor (generic name: omega-3-acid ethyl esters), also marketed by the name Lovaza, which is a preparation of EPA and DHA as ethyl esters [16]. The FDA-approved indication for this product is the reduction of triglycerides in patients having “very high” (>500 mg/dL) plasma triglyceride levels [17]. Following the approval of Omacor/Lovaza, several omega-3 products have been invented for this indication and are either on the market or under development.

LC omega-3 fatty acids occur naturally in fish oil mostly in the form of triglycerides. An alternative form of omega-3 s found in nature is as phospholipids, for which a major source is Antarctic krill (*Euphausia suberba*), an abundant krill species found in the Southern ocean. The main phospholipid in krill oil is phosphatidylcholine [18], an important source of the nutrient choline which since the 1930s has been known to have effects on how lipids, including cholesterol, are handled by the body [19]. Choline is oxidized to betaine, a methyl donor involved in the metabolism of homocysteine which at elevated concentrations has been associated with an increased risk of cardiovascular disease [20]. Therefore, phosphatidylcholine with a high content of omega-3 fatty acids may provide several components that potentially can have a positive impact on risk factors for developing cardiovascular disease.

There have been conflicting data as to whether LC-omega-3 fatty acids reduce, increase or leave circulating cholesterols unaffected. Early studies indicated clear and beneficial effects of these fatty acids, for example Illingworth et al. showed a significant decrease of both total cholesterol and LDL-c in healthy volunteers fed fish oil as compared to controls. However, in this study a very high LC omega-3 dose of 24 g/day was used [21]. More recent studies, applying lower omega-3 doses, have not been able to demonstrate similar effects on circulating cholesterols and for Omacor/Lovaza it has been published that in parallel with its triglyceride-reducing effect, a slight increase in LDL-c and high-density lipoprotein cholesterol (HDL-c) levels is observed [16, 22]. The general consensus in the literature seems to be that use of moderate doses of EPA and DHA, i.e., in the range of 0.5–5 g/day, does not reduce LDL-c concentrations in blood, a conclusion also reached by an Evidence-based Practice Center program under contract to the Agency for Healthcare Research and Quality (AHRQ), US Department of Health and Human Services in a report issued already back in 2004 [23] and updated recently with the same conclusion [24]. However, a remaining issue with the published data on LC omega-3 s and cholesterols is that important factors like dose levels, duration of treatment, type of omega-3 preparations used, EPA/DHA ratio in the dosed products, baseline cholesterol levels, diet, age and health status of the subjects as well as compliance with the planned dosing regimens vary considerably. It is therefore difficult to conclude uniformly on what effects an optimal and well-controlled use of an optimal preparation of omega-3 fatty acids could have on cholesterol levels in the most relevant patient population, i.e., in patients with pathologically elevated levels and thus having a significant risk of developing cardiovascular disease.

The primary objective of the study presented here was to undertake a first investigation of the long-term effects of a novel and highly purified phospholipid preparation rich in EPA and DHA, as well as choline, on blood levels of total cholesterol, LDL-c, HDL-c and triglycerides in dyslipidemic animals. The phospholipids in the preparation used in the study were extracted from krill oil. The animal model chosen for the study were cynomolgus monkeys with naturally occurring diabetes type-2 which, as a result of the disease, also had elevated blood lipids. The model is described in detail in [25] and has shown to be a relevant model for human disease also in other aspects of diabetes, like for the development of left ventricular systolic and diastolic dysfunction frequently seen in diabetic patients [26]. As secondary objectives, we also investigated if the preparation had effects on diabetes markers and markers of inflammation. The safety of the product was assessed by measuring liver, kidney and coagulation biomarkers. Plasma and erythrocyte levels of EPA and DHA were monitored at multiple occasions during treatment.

The data reported in this article originate from a study which also analyzed the effects of the phospholipid preparation on the time course of erythrocyte membrane levels of 24 different fatty acids and the time course in plasma of 6 endocannabinoid-type compounds being downstream metabolites of some of the fatty acids analyzed in the erythrocytes. The results of these analyses will be published separately.

## Results

### Plasma concentrations of EPA, DHA and DPA

**Fig. 1:**
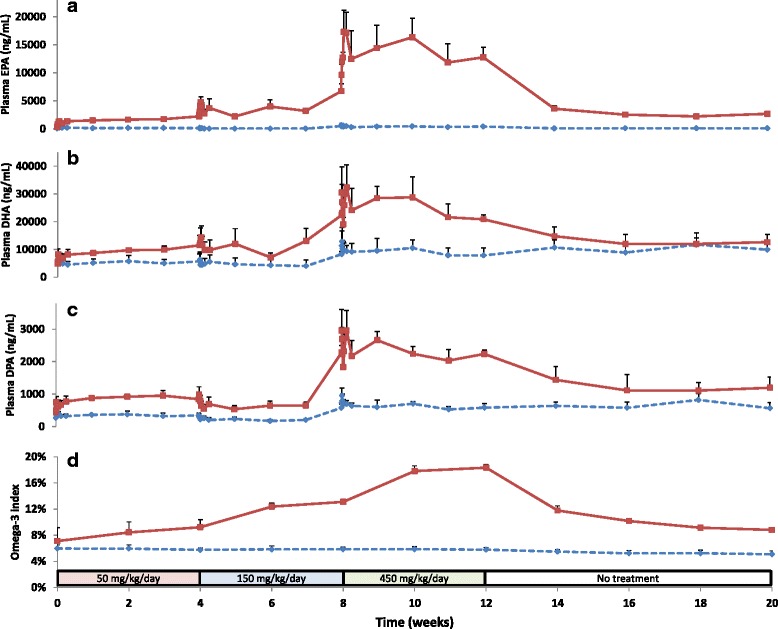
Mean concentrations of EPA (panel **a**), DHA (panel **b**) and DPA (panel **c**) in plasma and omega-3 index in erythrocytes (panel **d**) in the control (*dotted lines*) and treated (*unbroken lines*) groups during the 12-week treatment (weeks 0-12) followed by 8 weeks wash-out (weeks 12-20). Vertical lines indicate SEM for each data-point. Dose-levels at the bottom of the graph refer to phospholipid doses

Plasma samples analyzed for EPA and DHA showed that the concentration of these fatty acids increased with increasing dose (Fig. 1, panels a and b). The mean baseline concentrations of EPA and DHA in the treated group were 332 and 4843 ng/ml, respectively. After 4 weeks of treatment with the low dose of 50 mg/kg/day and just prior to the increase to the intermediate dose the mean plasma concentrations had increased to 2233 and 11477 ng/ml. Following completion of treatment with the intermediate dose of 150 mg/kg/day the mean plasma concentrations were 6727 and 22400 ng/ml, respectively, and at the end of the treatment with 450 mg/kg/day the concentrations were 12767 and 20867 ng/ml, respectively. At the end of the 8-week wash-out period, the EPA and DHA plasma concentrations were 2693 and 12627 ng/ml, respectively. For DPA, which might be considered a metabolite of EPA, the mean plasma concentration in the treated group was 444 ng/ml at baseline, 843 ng/ml at the end of the low dose treatment, 2277 at the end of the intermediate dose treatment, 2237 ng/ml at the end of the high dose treatment and 1196 ng/ml at the end of the wash-out period (Fig. 1, panel c). In the control group there was no change in the EPA concentration while an apparent, slight increase in DPA and DHA concentrations was noted during treatment, in particular during the period the highest dose was given (Fig. 1, panels a-c).

### Omega-3 index

The relative amount in percent of EPA + DHA in erythrocyte membranes as compared to the total amount of fatty acids in the membrane, the so-called omega-3 index [27], increased dose-dependently during the treatment period while no noticeable changes were observed in the control group (Fig. 1, panel d). At baseline the index was generally around 5–6% except for one animal which had an index of 11.2%. The mean baseline omega-3 index in the control group was 6.0% and in the treated group 7.1%. The lowest dose, 50 mg/kg/day, increased the index to a mean of 9.2% during the 4 weeks of dosing, the intermediate dose (150 mg/kg) increased it further to 13.1% and the highest dose (450 mg/kg/day) to 18.3% at the end of the 12-week dosing. Following cessation of dosing the index declined and after 8 weeks without treatment the mean omega-3 index was 8.8%. Interestingly, this rate of increase and decline is consistent with both EPA and DHA having standard first-order elimination kinetics.

Further details of the time course of the concentration of EPA, DHA and 22 other fatty acids in the erythrocytes during and after treatment will be published separately.

### Blood lipids and apolipoproteins

**Fig. 2:**
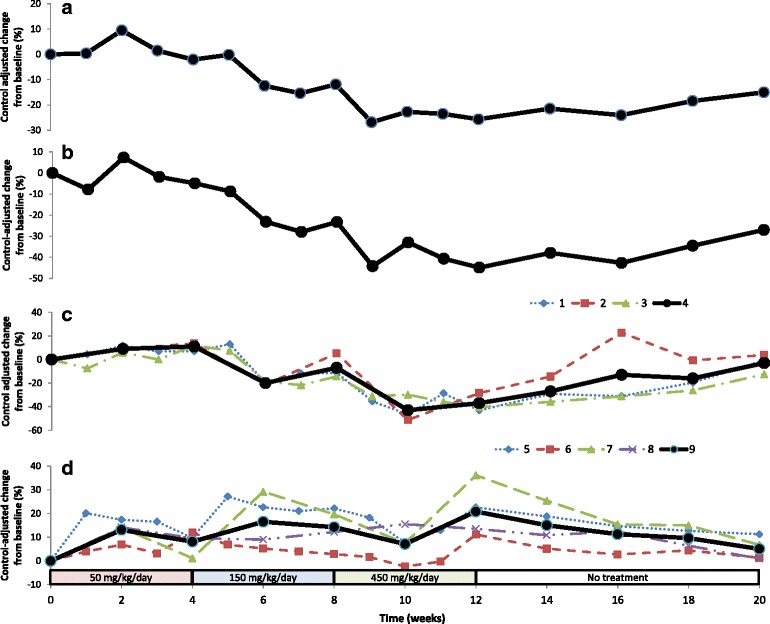
Panel **a**: Control-adjusted mean change in total serum cholesterol measured by Siemenes Advia-2400. Panel **b**: Control-adjusted mean change of calculated non-HDL cholesterol. Panel **c**: Control-adjusted mean change of LDL parameters assessed by 3 different methods. 1: LDL-c by selective chemically clearing method (Siemens Advia-2400); 2: LDL particles by NMR; 3: ApoB100 by turbidimetry (Siemens Advia-2400); 4: the mean of the three LDL methods. Panel **d**: Control-adjusted change of HDL parameters assessed by 4 different methods. 5: HDL-c by polymer/detergent method (Siemens Advia-2400); 6: HDL concentration (calculated) by NMR; 7: HDL particles by NMR; 8: ApoA1 by turbidimetry (Siemens Advia-2400); 9: The mean results obtained with the four HDL methods. Dose-levels at the bottom of the graph refer to phospholipid doses

**Fig. 3:**
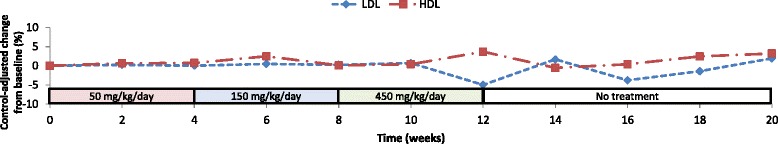
Control adjusted mean values for % change of LDL (*unbroken line*) and HDL (*dotted line*) particle size. Dose-levels at the bottom of the graph refer to phospholipid doses

The results for the primary parameters, the blood lipids and the apolipoproteins, are presented in Table 1. The concentrations of LDL were assessed in three different ways; LDL-c by the selective-chemically clearing method, total LDL particles by nuclear magnetic resonance (NMR) spectroscopy and ApoB100 by immune turbidimetry. Concentrations of HDL were assessed in four different ways; HDL-c by the polymer/detergent method and by NMR spectroscopy, total HDL particles by NMR spectroscopy and ApoA1 by immune turbidimetry. There was a marked variation in baseline values among the animals but for most of the primary parameters, higher-than-normal values were measured at start of treatment. The control-corrected values for the primary parameters are presented graphically in Fig. 2 and show that when treated with the intermediate and high dose levels of the phospholipid preparation, i.e., 150 and 450 mg/kg/day, total cholesterol, non-HDL cholesterol and LDL-c were dose-dependently reduced. In parallel to the reduction of LDL-c and LDL particles a reduction of ApoB100, the main apolipoprotein in LDL [28, 29], was observed. HDL-c and HDL particles, on the other hand, were increased during treatment. ApoA1, the principal apolipoprotein in HDL [30, 31], also increased although not to the same extent as HDL-c. The size of the LDL and HDL particles did not change as a result of treatment with the omega-3 rich phospholipid (Fig. 3). After cessation of treatment in week 12, both total cholesterol and LDL-c concentrations increased towards the pre-dose levels, while HDL-c concentrations decreased towards the pre-dose level. This rebound effect further indicates that the changes in the cholesterols are significant effects which are reversible after stopping the treatment and, thus, directly linked to the administration of the study preparation and the coherent changes in the omega-3 index. Plasma triglycerides were markedly reduced by treatment, most significantly after the highest dose of 450 mg/kg/day. Treatment did not seem to consistently affect the levels of apolipoproteins ApoB48 or ApoE, however the levels of ApoC3, an important apolipoprotein in VLDL [32, 33], was reduced during the treatment period although not in a dose-related manner (Table 2). These three apolipoproteins were not followed during the wash-out period after cessation of treatment.

**Table 1:** Mean and SEM values for the primary parameters total cholesterol, LDL assessed by three different methods, HDL assessed by four different methods, non-HDL-c (calculated), and triglycerides assessed by two different methods in serum from the NHPs at baseline, during and after treatment with the omega-3 rich phospholipid preparation. Dose levels of the phospholipids, EPA, DHA and choline are given at the end of the table

Parameter	Group	Value	Baseline	Week after start of treatment															
				1	2	3	4	5	6	7	8	9	10	11	12	14	16	18	20
Total cholesterol (mg/dL)	Control	Mean	139	138	139	141	143	144	138	142	156	147	142	145	149	153	169	164	172
		SEM	25.9	23.9	26.5	24.1	22.0	31.9	18.3	21.7	20.8	22.6	20.2	18.6	23.8	22.5	30.8	32.7	34.0
	Treated	Mean	173	172	192	176	176	177	147	148	176	137	140	143	140	155	178	182	196
		SEM	41.2	41.2	51.3	37.0	43.6	43.6	19.9	21.2	34.9	22.6	30.8	26.9	21.1	33.5	50.6	53.1	57.7
	Control adjusted^a^		0	0	9	1	−2	0	−12	−15	−12	−27	−23	−24	−26	−21	−24	−18	−15
LDL-c by Selective-chemically clearing method (mg/dL)	Control	Mean	57.7	57.5	54.5	54.1	53.1	54.8	55.9	50.9	60.3	61.6	65.6	59.0	65.9	67.0	71.9	71.0	66.6
		SEM	11.7	6.07	7.15	7.16	4.47	11.98	2.76	4.70	3.80	4.81	6.40	2.07	4.71	4.60	6.96	6.13	7.61
	Treated	Mean	85.2	91.6	95.8	87.8	87.4	91.9	64.1	61.9	81.1	61.9	48.1	66.3	64.1	75.8	87.7	94.5	98.7
		SEM	35.8	38.7	44.4	36.3	36.1	37.3	18.2	17.9	30.1	19.4	5.2	25.5	24.2	27.9	38.6	39.6	38.8
	Control adjusted^a^		0	4	12	7	7	13	−20	−12	−11	−35	−47	−29	−43	−29	−31	−20	−1
LDL particles by NMR (nmol/L)	Control	Mean	587	-	621	-	664	-	727	-	758	-	728	-	633	876	724	762	760
		SEM	175	-	184	-	258	-	258	-	223	-	219	-	130	239	184	220	221
	Treated	Mean	1085	-	1177	-	1229	-	828	-	1171	-	424	-	627	1000	1058	985	875
		SEM	612	-	614	-	633	-	325	-	455	-	247	-	300	423	444	423	259
	Control adjusted^a^		0		9		14		−21		5		−51		−28	−14	23	−1	4
ApoB100 by immune turbidimetry (g/L)	Control	Mean	0.37	0.36	0.36	0.39	0.37	0.38	0.38	0.41	0.46	0.47	0.52	0.54	0.55	0.58	0.63	0.61	0.60
		SEM	0.06	0.05	0.04	0.04	0.05	0.10	0.06	0.08	0.08	0.09	0.09	0.09	0.11	0.11	0.15	0.17	0.16
	Treated	Mean	0.52	0.50	0.57	0.56	0.64	0.55	0.43	0.45	0.58	0.48	0.57	0.59	0.55	0.62	0.72	0.74	0.78
		SEM	0.19	0.19	0.22	0.18	0.27	0.17	0.12	0.12	0.17	0.13	0.18	0.20	0.16	0.20	0.28	0.30	0.31
	Control adjusted^a^		0	−8	6	0	12	7	−19	−22	−14	−31	−30	−36	−40	−36	−31	−26	−13
HDL-c by Polymer-Detergent method (mg/dL)	Control	Mean	83.8	86.2	86.1	88.7	90.1	94.6	88.4	96.7	106	99.9	93.5	100	102	106	122	119	121
		SEM	12.2	13.6	12.3	11.6	16.0	27.1	14.0	21.5	22.1	25.9	17.6	21.9	26.6	23.5	35.3	37.7	38.2
	Treated	Mean	124	118	138	124	127	123	93.3	97.4	122	86.9	100	96.0	88.3	105	130	136	146
		SEM	46.2	45.5	53.5	38.7	45.5	43.9	20.8	22.1	33.7	23.1	41.5	31.5	23.8	35.6	53.6	56.9	61.3
	Control adjusted^a^		0	20	17	17	10	27	23	21	22	18	−13	13	23	19	15	13	11
HDL concentration (calculated) by NMR (mg/dL)	Control	Mean	67.3	-	66.3	-	65.3	-	60.3	-	56.3	-	58.3	-	56.3	56.3	58.7	58.7	63.3
		SEM	13.3	-	18.0	-	17.7	-	15.9	-	11.3	-	10.3	-	13.6	11.6	14.3	14.7	14.0
	Treated	Mean	62.0	-	66.0	-	58.3	-	69.7	-	62.0	-	56.0	-	72.0	64.7	61.7	62.0	61.0
		SEM	11.5	-	7.81	-	8.25	-	8.17	-	5.03	-	18.1	-	9.64	5.67	7.80	9.64	8.62
	Control adjusted^a^		0		14		1		29		20		8		36	25	15	15	7
HDL particles by NMR (μmol/L)	Control	Mean	36.7	-	34.9	-	34.8	-	33.5	-	32.7	-	34.5	-	35.3	34.2	36.9	37.4	39.7
		SEM	6.46	-	5.66	-	5.49	-	6.00	-	4.85	-	4.53	-	4.61	4.75	6.03	5.32	4.75
	Treated	Mean	35.0	-	38.3	-	36.7	-	35.1	-	35.8	-	40.3	-	39.0	36.7	39.6	38.2	38.8
		SEM	1.31	-	1.19	-	1.33	-	2.08	-	2.21	-	4.33	-	2.27	1.41	1.79	1.20	1.19
	Control adjusted^a^		0		14		10		9		12		16		14	11	12	6	1
ApoA1 by immune turbidimetry (g/L)	Control	Mean	0.98	0.93	0.96	0.93	0.97	0.88	0.94	0.95	0.99	0.94	0.94	0.90	0.90	0.93	0.90	1.24	1.34
		SEM	0.08	0.08	0.09	0.06	0.08	0.08	0.08	0.08	0.07	0.07	0.06	0.05	0.06	0.07	0.06	0.24	0.25
	Treated	Mean	0.98	0.97	1.02	0.96	1.08	0.94	0.99	0.98	1.02	0.95	0.82	0.90	1.01	0.98	0.93	1.27	1.35
		SEM	0.05	0.03	0.04	0.02	0.10	0.03	0.04	0.03	0.05	0.02	0.13	0.04	0.05	0.03	0.03	0.12	0.14
	Control adjusted^a^		0	4	7	3	12	7	5	4	3	2	−2	0	11	5	3	4	1
Non-HDL-c (calculated) (mg/dL)	Control	Mean	83.8	86.2	86.1	88.7	90.1	94.6	88.4	96.7	106.2	99.9	93.5	100	102	106	122	119	121
		SEM	12.2	13.6	12.3	11.6	16.0	27.1	14.0	21.5	22.1	25.9	17.6	21.9	26.6	23.5	35.3	37.7	38.2
	Treated	Mean	124	118	138	124	127	123	93.3	97.4	122	86.9	100	96.0	88.3	105	130	136	146
		SEM	46.2	45.5	53.5	38.7	45.5	43.9	20.8	22.1	33.7	23.1	41.5	31.5	23.8	35.6	53.6	56.9	61.3
	Control adjusted^a^		0	−8	7	−2	−5	−9	−23	−28	−23	−44	−33	−41	−45	−38	−43	−34	−27
Triglycerides by enzymic method (GK-PK-LDH) (mg/dL)	Control	Mean	99.8	133	123	143	174	174	141	194	213	200	164	210	208	210	239	267	312
		SEM	57.3	95.4	85.4	104	127	137	88.3	143	156	149	111	153	168	150	192	217	262
	Treated	Mean	211	138	225	182	239	129	123	125	188	107	192	123	93	165	231	262	267
		SEM	98.5	71.9	102	72.5	103	51.6	40.3	39.7	66.3	42.2	134	59.0	24.7	72.2	126	146	156
	Control adjusted^a^		0	−46	−5	−17	−20	−53	−49	−61	−40	−90	−58	−92	−74	−48	−28	−46	−69
Triglycerides (calculated) by NMR (mg/dL)	Control	Mean	79.0	-	101	-	151	-	119	-	174	-	141	-	172	178	221	211	256
		SEM	30.0	-	45.2	-	93.0	-	65.8	-	116	-	88.7	-	120	119	166	157	200
	Treated	Mean	166	-	158	-	184	-	87	-	131	-	105	-	65.3	127	190	196	220
		SEM	63.6	-	56.5	-	69.4	-	20.6	-	41.0	-	64.2	-	10.0	52.2	103	101	129
	Control adjusted^a^		0		−32		−80		−98		−142		−115		−178	−149	−166	−149	−192
Dose (mg phospholipids/kg bw/day)				50				150				450				0			
Dose (mg [EPA/DHA]/kg bw/day)				9.35/5.48				28.0/16.4				84.1/49.3				0/0			
Dose (mg choline/kg bw/day)				5.83				17.5				52.5				0			

- no sample

^a^ % change of treated group relative to control group

**Table 2:** Mean and SEM values and control adjusted changes for the apolipoproteins ApoB48, ApoC3 and ApoE in serum from the NHPs at baseline and during treatment with the omega-3 rich phospholipid preparation. Dose levels of the administered phospholipids, EPA, DHA and choline are given at the end of the table

Parameter	Group	Value	Baseline	Week after start of treatment											
				1	2	3	4	5	6	7	8	9	10	11	12
ApoB48 (mg/dL)	Control	Mean	1.84	1.86	1.96	1.77	1.84	1.84	1.97	1.96	1.84	2.00	1.95	1.81	1.93
		SEM	0.06	0.05	0.04	0.17	0.12	0.20	0.06	0.07	0.15	0.05	0.08	0.12	0.16
	Treated	Mean	2.19	2.42	2.17	2.23	2.37	2.25	2.39	2.42	2.27	2.47	2.22	2.61	2.60
		SEM	0.21	0.12	0.31	0.05	0.30	0.19	0.31	0.20	0.15	0.07	0.37	0.15	0.10
	Control adjusted^a^		-	10	−8	7	8	3	1	4	4	5	−7	22	15
ApoC3 (ng/mL)	Control	Mean	50.7	62.3	42.5	37.0	40.9	46.2	48.2	39.9	56.9	44.2	28.9	42.2	38.0
		SEM	14.5	10.1	3.17	5.40	10.9	15.1	12.0	17.9	18.3	11.4	11.9	17.9	20.4
	Treated	Mean	45.8	33.6	31.3	21.2	21.4	18.7	18.2	13.4	40.7	28.0	30.2	12.5	14.1
		SEM	21.9	20.0	18.7	13.2	13.8	10.5	7.70	10.9	21.0	13.4	21.7	4.85	5.42
	Control adjusted^a^		-	−71	−34	−40	−44	−57	−56	−51	−26	−34	−1	−45	−31
ApoE (ng/mL)	Control	Mean	1.06	1.63	1.20	1.23	1.26	1.99	1.15	1.13	1.36	1.36	1.29	1.59	1.32
		SEM	0.12	0.24	0.19	0.27	0.07	0.40	0.18	0.10	0.19	0.09	0.17	0.15	0.05
	Treated	Mean	1.68	1.84	1.72	1.44	2.12	2.37	1.95	2.72	1.73	2.61	2.88	2.41	2.67
		SEM	0.19	0.34	0.27	0.14	0.28	0.62	0.36	0.57	0.30	0.62	0.99	0.50	0.65
	Control adjusted^a^		0	−42	−10	−30	6	−46	10	54	−25	23	70	4	29
Dose (mg phospholipids/kg bw/day)				50				150				450			
Dose (mg [EPA/DHA]/kg bw/day)				9.35/5.48				28.0/16.4				84.1/49.3			
Dose (mg choline/kg bw/day)				5.83				17.50				52.50			

^a^ % change of treated group relative to control group

### Biomarkers of diabetes and inflammation

The treatment with the purified phospholipids had no noticeable effect on the diabetes-related parameters glycated hemoglobin (HbA1c), glucose and insulin (Table 3), which is in line with findings in a meta-analysis of human studies on effects of omega-3 fatty acids on diabetes type-2 [34]. Neither was there any effect on the inflammation markers (data shown in Additional file 1), however the fact that the C-reactive protein (C-rp) levels were low at baseline and throughout the study indicated that the animals had no on-going systemic inflammation, therefore this finding was as expected. Having an on-going inflammation was not one of the inclusion criteria, neither were the animals screened pre-study with respect to inflammation.

**Table 3:** Mean and SEM values for the monitored diabetes-related parameters Hb1Ac, glucose and insulin in blood from the NHPs at baseline and during treatment with the omega-3 rich phospholipids. Dose levels of administered phospholipids, EPA, DHA and choline are given at the end of the table

Parameter	Group	Value	Baseline	Week after start of treatment					
				2	4	6	8	10	12
Hb1Ac (%)	Control	Mean	9.07	8.60	9.00	9.00	9.37	9.43	9.80
		SEM	2.31	2.05	2.35	2.40	2.56	2.69	2.87
	Treated	Mean	6.63	6.53	6.50	6.40	6.50	6.40	6.67
		SEM	1.43	1.64	1.46	1.37	1.23	1.48	1.49
Glucose (mg(dL)	Control	Mean	195	197	196	182	177	181	203
		SEM	64.1	71.8	62.0	61.4	65.8	64.5	71.9
	Treated	Mean	148	135	157	127	158	119	141
		SEM	41.2	31.7	38.3	35.5	30.0	20.0	32.3
Insulin (mIU/mL)	Control	Mean	33.1	26.3	21.9	46.2	20.9	21.8	28.1
		SEM	5.27	5.32	4.86	21.28	5.51	5.61	10.5
	Treated	Mean	66.7	112	104	91.6	115	74.2	96.7
		SEM	29.6	52.1	44.3	40.7	60.5	29.0	54.1
Dose (mg phospholipids/kg bw/day)				50		150		450	
Dose (mg [EPA/DHA]/kg bw/day)				9,35/5,48		28,1/16,4		84.2/49.3	
Dose (mg choline/kg bw/day)				5,83		17,5		52,5	

### Biomarkers of safety

The results from analysis of the parameters indicative of effects on liver function, kidney function and coagulation are listed in Table 4 and show that the 12-week treatment with the phospholipids up to 450 mg/kg/day did not impact any of the liver and kidney biomarkers, and, furthermore, had no effects on the coagulation parameters monitored. All hematology parameters remained unchanged throughout the 12-week treatment period (data shown in Additional file 1). The bodyweight of all animals was unchanged during the 20 weeks the study lasted (data shown in Additional file 1).

**Table 4:** Mean and SEM values for the safety parameters measured in the control and treated groups at baseline and during the 12 week treatment period. Dose levels of phospholipids, EPA, DHA and choline are given at the end of the table

Parameter	Group	Value	Baseline	Week after start of treatment		
				4	8	12
Alanine transaminase (ALT) (U/L)	Control	Mean	128	127	182	101
		SEM	59	54	103	32
	Treated	Mean	86	87	65	75
		SEM	50	24	2	14
Aspartate Aminotransferase (AST) (U/L)	Control	Mean	36	32	44	33
		SEM	6	4	13	4
	Treated	Mean	32	27	30	42
		SEM	9	5	6	11
Alkaline Phosphatase (ALP) (U/L)	Control	Mean	225	221	226	220
		SEM	37	40	46	56
	Treated	Mean	162	166	140	169
		SEM	37	44	36	68
Blood Urea Nitrogen (BUN) (mmol/L)	Control	Mean	6.69	6.13	6.46	6.29
		SEM	0.46	0.69	0.67	0.69
	Treated	Mean	6.16	5.65	5.95	5.65
		SEM	0.04	0.52	0.63	0.54
Creatinine (μmol/L)	Control	Mean	62	64	63	61
		SEM	23	23	23	23
	Treated	Mean	77	74	80	76
		SEM	5.2	5.6	5.3	5.4
Uric acid (μmol/L)	Control	Mean	6.07	4.90	5.37	6.40
		SEM	0.17	0.12	0.12	0.25
	Treated	Mean	7.00	9.17	6.70	7.13
		SEM	1.04	2.99	1.17	1.33
Cystatin C (mg/L)	Control	Mean	1.23	1.17	1.15	1.10
		SEM	0.21	0.23	0.22	0.16
	Treated	Mean	1.21	1.23	1.26	1.24
		SEM	0.01	0.10	0.10	0.10
Prothromin time (PT) (sec)	Control	Mean	9.90	9.8	10.5	10.6
		SEM	0.36	0.40	0.52	0.64
	Treated	Mean	10.1	10.0	10.6	10.8
		SEM	0.29	0.36	0.32	0.20
Activated Partial Thromboplastin time (APTT) (sec)	Control	Mean	19.3	19.7	19.1	18.9
		SEM	0.18	0.47	0.73	1.22
	Treated	Mean	21.0	21.9	21.5	21.5
		SEM	1.93	1.85	2.18	2.55
Thrombin Time (TT) (sec)	Control	Mean	30.5	29.8	27.9	26.8
		SEM	0.52	1.73	1.54	2.18
	Treated	Mean	29.3	28.8	28.7	28.1
		SEM	0.92	2.80	1.91	1.34
Dose (mg phospholipids/kg bw/day)				50	150	450
Dose (mg [EPA/DHA]/kg bw/day)				9.35/5.48	28.1/16.4	84.2/49.3
Dose (mg choline/kg bw/day)				5.83	17.5	52.5

## Discussion

A large number of animal studies on effects of omega-3 fatty acids and other treatment regimens on elevated blood lipids have been published. The specifics of the lipid biochemistry in the various species are important to consider when studies on this topic in animal models are designed. Commonly used species in this type of research are mice, rats, dogs and pigs, but it is worth noting that none of these species has a blood lipid profile similar to the profile seen in humans, one important reason being that these species lack or have very low levels of the cholesteryl-ester transferring protein (CETP), an enzyme that transfers cholesteryl esters from HDL to LDL and VLDL [35]. Therefore these species have very low levels of LDL-c. Species that do express CETP, and therefore LDL-c levels more similar to humans, include rabbits, hamsters and non-human primates. According to Yin et al. (2012), who thoroughly reviewed the relevance of the use of various species in dyslipidemia research, the animal species most likely to predict effects of lipid-lowering treatment in dyslipidemic humans is the non-human primate and in particular the rhesus and cynomolgus strains [36]. The use of non-human primates in research should be stringently justified due to their high level of intelligence and sentience [37] but in order to extract the most predictive and relevant information from the current study we decided to use the cynomolgus monkey as our animal model. Furthermore, the animals included were suffering from naturally occurring diabetes type-2, although of such a mild type that treatment by glucose-lowering therapies was not necessary. Using animals that were hyperlipidemic because of a naturally occurring disease was considered more relevant than a model where the dyslipidemia was induced artificially, e.g., by subchronic feeding with a high fat or high sucrose diet. However, because of this special selection of animal model, the number of primates included was deliberately kept to a minimum in accordance with the 3Rs and as recommended in the Weatherall report on the use of non-human primates in research [38]. For the animals included, care and use were conducted in accordance with all applicable assessments and accreditations of the laboratory animal care (AAALAC) regulations and guidelines and the institutional animal care and use committee approved all applied procedures. Furthermore, the animals were not euthanized at the end of the 20-week in-life phase of the study but were forwarded into a further wash-out period with the intention of being used for other studies later. As a means to compensate for the low number of animals per group and increase the reliability of the results, the most important parameters were measured by more than one method where possible. This was the case for LDL and HDL as well as for triglycerides.

The main finding in the study presented here was that the purified, LC omega-3 rich phospholipid preparation extracted from krill oil reduced total circulating cholesterol, LDL-cholesterol and triglycerides and increased HDL-cholesterol when dosed over a 12-week period to dyslipidemic non-human primates with naturally occurring diabetes type-2. The animals were given escalating doses of the test and control articles, with each dose level given daily for 4 weeks. The observed effects were mostly dose-dependent although the lowest dose, which was 50 mg phospholipids/kg/day and given during the first 4 weeks of treatment, had no detectable effect on total cholesterol and LDL-c, however an apparent increase in HDL and ApoA1 was observed. Triglyceride levels were also reduced. The lack of effect on total cholesterol and LDL-c with the lowest dose could either be due to the dose being too low or that treatment with this preparation needs to go on for longer than 4 weeks to have an effect on these parameters. The intermediate dose of 150 mg/kg/day, given during weeks 4–8, clearly reduced total cholesterol, LDL-c and triglycerides and increased HDL-c and the highest dose of 450 mg/kg/day, given during weeks 8–12 of treatment, had even more significant effects on these primary parameters and proved the potential of this preparation to modify blood levels of the cholesterols in a health-beneficial pattern. When judging these findings, however, it is important to keep the study design in mind, particularly the fact that all three doses were given to the same animals and that there was a possible accumulation effect. Hence, the effects observed with the intermediate dose may have been influenced by the low dose and likewise, the effects of the high dose may have been influenced by the low and intermediate doses. From this it is evident that although the data indicated a dose–response relationship for most parameters, this relationship should be interpreted with some caution.

In blood, cholesterol is mostly associated with plasma lipoproteins, which allow transport around in the body at concentrations much higher than would otherwise be possible for lipophilic compounds. The specific lipoproteins LDL and HDL are built around one major apolipoprotein each so the concentration of these apolipoproteins is generally correlated with the concentrations of the associated lipoproteins, although variation in size of the lipoprotein particles can to some degree alter the correlation between concentration of apolipoprotein and the associated cholesterol [39]. In the present study, however, LDL and HDL particle size were measured and it was observed that the particles did not change their size during the course of the study. The measurements of the main apolipoproteins for LDL (ApoB100) and HDL (ApoA1) revealed that the patterns for LDL and ApoB100 were similar, and the same was the case for HDL and ApoA1. These coherent patterns strengthen the evidence that the observed reduction in total cholesterol and LDL-c and the increase in HDL-c are biologically significant.

In a review published in 2006, Balk and co-workers evaluated 21 studies on effects of fish oil and plant-source omega-3 fatty acids on serum cholesterol and other markers considered to be risk factors for development of cardiovascular disease [40]. By combining the data in the 21 studies they found clear and dose-related effects of fish-oil only on triglyceride levels while there was no effect on total cholesterol. HDL-c and LDL-c increased slightly. The data for omega-3 from plants, i.e., alpha-linolenic acid (C18:3n3), were more unclear and no conclusive evidence of effects on the serum biomarkers was seen. In a meta-analysis published in 2012 and based on 20 studies including 68 680 patients, Rizos et al. concluded that LC omega-3 PUFAs are not statistically significantly associated with major cardiovascular outcomes across various patient populations [41]. This publication did not specify the source of the omega-3 s administered to the populations but given that the vast majority of omega-3 products on the market originate from fatty fish it can be assumed that most of the subjects used fish oil. There is a lack of larger studies and meta-analyses of effects of krill oil and results from individual studies are equivocal with respect to effects on cholesterols. Bunea at al. gave Neptune Krill oil (NKO) to patients with hyperlipidemia at doses of 1–3 g/day for 90 days and observed dose-dependent effects on all main blood lipids. The highest dose of 3 g/day reduced total cholesterol by 18%, LDL-c by 37% and triglycerides by 28% and at the same time increased HDL-c by as much as 55% [42]. In a recently published randomized controlled clinical trial, Lobraico and co-workers gave krill oil containing 1 g omega-3 fatty acids to patients with diabetes type-2 for 17 weeks and demonstrated that following treatment, the patients had significantly improved endothelial function and increased HDL but no effect was seen on LDL levels, neither relative to baseline nor to a control population given olive oil [43]. Berge et al. (2014), giving krill oil up to 4 g/day for 12 weeks to subjects with borderline high or high serum triglyceride levels did not see any significant effects on cholesterols but observed a reduction in triglycerides [44]. Interestingly, a recent randomized, cross-over study from Cicero et al. compared the effects of 500 mg krill oil given twice a day with those of 1000 mg omega-3 ethyl ester twice a day and found that although the ethyl esters were more efficacious than krill oil in reducing triglycerides, only krill oil was able to significantly increase HDL-c and ApoA1 levels [45]. One reason for this difference in outcome might be the baseline level of blood lipids in the studied populations because generally, the more abnormal baseline levels of blood lipids the more significant effects might be expected of the various lipid-lowering treatments.

The main components of normal krill oil are around 40% phospholipids in addition to triglycerides, free fatty acids and the natural antioxidant astaxanthin [46]. The major part (>65%) of the omega-3 fatty acids in krill oil is in the phospholipid fraction. Fish oil consists mainly of triglycerides with which the omega-3 fatty acids are associated. This variation in omega-3 fatty acid chemistry might cause a difference in important factors mediating effects on blood lipids like distribution to tissues, distribution among the various lipoproteins in blood and effects on regulators of lipid metabolism in the liver. In addition, the main phospholipid in krill oil is phosphatidylcholine which is the main source of the essential nutrient choline in mammals. Choline has been shown to influence cholesterol metabolism and transport [47] and the administration of a combination of omega-3 fatty acids and choline could potentially cause an additive or synergistic, beneficial effect on the blood cholesterol levels. Hence, when considering pharmacological effects of LC omega-3 s it is necessary to specify the source and chemical composition of the fatty acids administered. In the present study, the omega-3 s were bound only in phospholipids and this is a preparation basically different from other forms of omega-3 s that have been investigated previously for similar indications and it should not be considered equivalent to natural krill oil.

The main conclusions from this work were based on control-corrected values, where the original measurements first were baseline-adjusted. This was done to take full value of the data obtained in the control group which was equal in size to the treated group. The control group was given the same vehicles as the treated group and is therefore expected to correct for any biological effects of the vehicles, ethanol and PEG400, as well as for effects of the frequent handling (dosing, weighing and blood sampling) of the animals during the course of the study. However, when considering the absolute values obtained during the analysis of the blood samples, the trends of reduced total cholesterol, LDL-c and triglycerides and increased HDL-c were obvious even without adjusting for the changes in the control animals but the dose–response relationships were more obvious when taking the control data into account, indicating that this could be the more relevant way to interpret the data.

The most important limitation of the current study is obviously the number of animals being included in the experiments. While a group size of 3 animals allows trends to be detected, statistical analyses are not meaningful with this low number. Therefore, the data were mainly used to assess trends in the various parameters and statistics was limited to estimating standard error of means in order to indicate the variation around the mean values.

## Conclusions

This study demonstrated that long-term treatment with a novel and highly purified omega-3 rich phospholipid preparation extracted from krill oil altered the blood lipid profiles in dyslipidemic, diabetic non-human primates markedly and in a health-beneficial manner by reducing total cholesterol, LDL-cholesterol and triglycerides while it increased HDL-cholesterol. No effects were observed on lipoprotein particle size and diabetes parameters. There were no effects on coagulation parameters, and commonly used biomarkers of liver and kidney toxicity were unchanged. This preparation may potentially be a useful therapy to normalize dyslipidemia without significant side-effects, either alone as a single-agent treatment or in combination with other therapies like statins or PCSK9 inhibitors. In addition, the known anti-inflammatory effects of the LC omega-3 fatty acids EPA and DHA could further reduce the risk of developing atherosclerotic, cardiovascular diseases. However, further pre-clinical studies are required to elucidate the full potential and the mechanism(s) of action of this preparation and to justify its entrance into clinical trials.

## Methods

### Test and control articles

The test article used was a preparation of 98% pure phospholipids, extracted from krill oil originating from Antarctic krill (*Euphausia superba*) supplied by Aker Biomarine, Oslo, Norway and formulated for oral administration by adding 12.5% poly-ethylene glycol with a MW of 400 (PEG400) and 3.5% absolute ethanol, hence the test article contained 84% (840 mg/g) phospholipids. Phospholipids from krill oil are rich in omega-3 fatty acids and the final preparation contained 157 mg EPA (C20:5n3)/g and 92 mg DHA (C22:6n3)/g. Gas-chromatography analysis detected 23 fatty acids in the end-product in addition to EPA and DHA, five of these were present with an abundance of more than 1% (10 mg/g): myristic acid (C14:0) 1.1%, palmitic acid (C16:0) 13.2%, oleic acid (C18:1n9) 2.1%, cis-vaccenic acid (C18:1n11) 3.1% and stearidonic acid (C18:4n3) 1.3%. Phosphatidylcholine comprised 90% of all phospholipids in the preparation, and the choline moiety constitutes 13% of the phosphatidylcholine molecule. Dose levels given throughout the text refer to the dose of phospholipids, i.e., a dose of 50 mg/kg/day means 50 mg of purified phospholipids/kg body weight/day. Daily doses of EPA, DHA and choline are however specified in the tables presenting the results. The control article was composed of 84% water, 12.5% PEG400 and 3.5% ethanol and by this, both the control and treated animals received the same dose of the vehicles but for the controls, the phospholipids were substituted by water. To ease the administration, the test article was warmed to ca. 35 °C and mixed with water just prior to dosing to make an aqueous emulsion with low viscosity. To keep consistency, the control article was treated identical to the test article. The phospholipid formulation used has been proven stable for at least 6 months.

### Animals

Six type-2 diabetic and dyslipidemic cynomolgus monkeys (*Macaca fascicularis*) were selected for the study, after screening a total of 34 animals. Inclusion criteria were predefined and were related to diabetes parameters (HbA1c, glucose and insulin) and blood lipid levels (triglycerides, total cholesterol, LDL-c and HDL-c). The animals included were defined diabetic and hyperlipidemic with reference to normal levels in this strain of monkeys housed at Crown Biosciences in Taicang, China. The vital data and the individual screening values for the monkeys included in the study are detailed in Table 5.

**Table 5:** Vital data and results from the screening of the animals included in the study. The normal range values refer to values measured in normal, non-diabetic cynomolgus monkeys housed at the laboratory where this study was conducted

Group	Animal ID	Sex	Age (years)	Bodyweight (kg)	Screening parameters						
					TC	LDL	HDL	TG	Hb1Ac	Glucose	Insulin
					(mg/dL)	(mg/dL)	(mg/dL)	(mg/dL)	(%)	(mg/dL)	(mIU/L)
Control	Control no. 1	M	14	9.22	140	57.0	57.0	162	13.8	261	9.70
	Control no. 2	M	15	10.0	103	48.0	38.0	55.0	10.2	234	22.4
	Control no. 3	F	13	4.32	191	76.0	80.0	89.0	4.6	67	36.7
Treated	Treated no. 1	M	14	8.30	188	74.0	60.0	269	17.5	157	200
	Treated no. 2	F	21	7.34	256	162	40.0	400	10.1	224	56.1
	Treated no. 3	M	20	10.8	128	49.0	61.0	50.0	5.90	109	51.8
Normal range					97 ± 4	35 ± 3	30–55	14–88	≤5	40–75	20–50

The animals were housed individually in cages, in rooms with a temperature of 20–23 °C, humidity 40–70% and a 12 h dark/light cycle. They were fed a standard monkey diet containing crude protein (≥16%), crude fat (≥4%), moisture (≤10%), ash (≤7%), fiber (≤4%), calcium (0.8–1.2%) and phosphorus (0.6–0.8%). The content of omega-3 fatty acids in the diet was not measured by the supplier (Beijing Keao Xieli Feed Co. Ltd., Beijing, P.R. China) and was therefore unknown. Care and use of the animals were conducted in accordance with all applicable assessments and accreditations of the laboratory animal care (AAALAC) regulations and guidelines. Crown bioscience institutional animal care and use committee (IACUC) approved all animal procedures used in the study. All the procedures related to handling, care and treatment of the animals were performed according to the guidelines approved by AAALAC. After each handling (weighing, bleeding or dosing), the animals were observed until they were able to stand up and alert if they were anesthetized. At the time of routine monitoring, the animals were checked for any effects of the compound on their behavior such as mobility, food and water consumption, body weight gain/loss, and any other abnormal activities. Clinical abnormalities observed were recorded and reported. After completion of the study the animals were returned to the stock of diabetic monkeys held at Crown Biosciences for a recovery period and following this they were allowed to be used in other studies.

### Administration of test and control articles

The control and test articles were given by gavage, 1 h after the first feeding of the day. The control article was administered at the same volume/kg as the test article. For the first 4 weeks, a phospholipid dose of 50 mg/kg/day was given, the next 4 weeks the dose was 150 mg/kg/day and for the last 4 weeks 450 mg/kg/day. Dosing volumes were based on body weights recorded bi-weekly after overnight fasting. When converted to human equivalent dose (HED) these doses were equivalent to approximately 1, 3 and 10 g/day, respectively, to an adult person of 70 kg.

The dosing was done by inserting a naso-/oral-/gastric tube into the stomach and injecting the correct volume as a bolus. The tube was then flushed with 5 mL lukewarm water to assure the administration of the entire volume.

Following completion of the 12-week dosing period, the animals were followed for 8 more weeks to investigate how selected parameters developed upon cessation of treatment. During these 8 weeks, neither the control nor the test article was given but blood samples were drawn bi-weekly and prepared for analysis.

### Blood sampling and analysis

After overnight fasting and before dosing, blood was sampled at baseline, i.e., before starting the treatment with the control or test item, and either weekly, bi-weekly or every 4 weeks during the 12-week dosing period for analysis of the experimental parameters. Weekly, the following primary parameters were analyzed in blood serum by a Siemens Advia-2400 Clinical Chemical System: Total cholesterol, LDL cholesterol (LDL-c), HDL cholesterol (HDL-c), triglycerides, apolipoprotein B100 (ApoB100), apolipoprotein A1 (ApoA1), apolipoprotein B48 (ApoB48), apolipoprotein C3 (ApoC3) and apolipoprotein E (ApoE). Non-HDL cholesterol (Non-HDL-c) was estimated by subtracting HDL-c from total cholesterol. Parameters analyzed bi-weekly in blood: Glycated hemoglobin (Hb1Ac, by ion-exchange HPLC), glucose (by Siemens Advia-2400), insulin (by Siemens Advia Centaur XP) and C-reactive protein (C-rp, by Siemens Advia-2400). Triglycerides and lipoproteins, including concentration and size of LDL and HDL particles, were measured bi-weekly in blood plasma by nuclear magnetic resonance (NMR) spectroscopy. The following parameters were measured bi-weekly in plasma by Luminex kits: soluble intercellular adhesion molecule-1 (sICAM-1), soluble vascular adhesion molecule-1 (sVCAM-1), P-selectin, monocyte chemotactic protein-1 (MCP-1) and macrophage colony-stimulating factor (M-CSF). Fatty acids in erythrocyte membranes, including the omega-3 index [27] were also measured bi-weekly by a previously described method based on formation of fatty acid methyl esters and analysis by gas chromatography with flame ionization detection [48] (only the omega-3 index values are reported here, the time-course of the 24 individual fatty acids analyzed is reported elsewhere). At baseline and every 4 weeks during the dosing period, just before increasing the dose to the next level, the following parameters were measured: aspartate aminotransferase (AST), alkaline phosphatase (ALT), blood urea nitrogen (BUN), creatinine, uric acid (UA), cystatin-C, prothrombin time (PT), activated prothrombin time (APTT), thrombin time (TT), white blood cell concentration (WBC, total and differential counting), red blood cell concentration (RBC), hematocrit (Hct), mean cellular volume of RBC (MCV), mean cellular hematocrit (MCH) and mean corpuscular hemoglobin concentration (MCHC).

Plasma sampled at baseline and at 1, 2, 4, 8, 12, 24 and 48 h and 7, 14 and 21 days after the first dose of each dose level, and in addition at 28 days after the first dose of the highest dose level of 450 mg/kg/day, were analyzed for the determination of EPA, DPA and DHA concentrations by a liquid chromatography tandem mass-spectrometry (LC-MS/MS) method specifically developed for this purpose. This method will be published separately but in brief, plasma samples were prepared using protein precipitation followed by alkaline hydrolysis to release the fatty acids from phospholipids, triglycerides and other lipid classes to the free form. Deuterated EPA (EPA-d5) and DHA (DHA-d5) were used as internal standards. The determination of fatty acid concentrations, which were the sum of endogenous EPA, DPA and DHA and these fatty acids stemming from the administered compound, was done by MS/MS (PE Sciex API 4000) using Turbolonspray in negative ion, multiple reaction monitoring mode.

The blood samples taken during the 8 weeks after end of treatment were analyzed for the following parameters: total cholesterol, LDL-c, HDL-c, triglycerides, apoB100, ApoA1, LDL particle size as well as omega-3 index and plasma concentrations of EPA, DPA and DHA. The methods applied were the same as listed above.

In addition to the above parameters, blood samples were taken bi-weekly for analysis of the following endocannabinoids and endocannabinoid-type compounds in plasma: anandamide, 2-arachidonoylglyceride, EPA ethanolamide, DHA ethanolamide, palmitoyl ethanolamide and oleoyl ethanolamide. The results of these analyses will be published separately, together with the results of the fatty acid concentrations in erythrocyte mebranes.

### Data presentation and statistics

The data tabulated for the main parameters (cholesterols, apolipoproteins and triglycerides) are firstly absolute arithmetic mean of each parameter in the control and treated group, with standard error of the mean (SEM) to indicate the variability in the groups, secondly the control adjusted changes. Control adjusted values were calculated by normalizing all values measured during and after treatment to the baseline values, i.e., by defining the baseline value in each animal as 100% and then calculate the change relative to baseline. The resulting mean at each time-point in the treated group is then subtracted from the mean of corresponding time-points in the control group, giving the control-adjusted change at each data-point. For diabetes and safety parameters, mean and SEM values are presented.

Given the low number of animals in each of the two groups, comparison of effects based on statistical criteria was not done. Despite the low number of animals in each group, arithmetic mean rather than median was used to describe the group changes in the measured parameters at each time-point, both for absolute values and for values normalized to baseline.

## Additional file

Additional file 1: Inflamm Hematol BW. (XLSX 28 kb)

## Abbreviations

ApoA1: Apolipoprotein A1
ApoB100: Apolipoprotein B100
ApoB48: Apolipoprotein B48
ApoC3: Apolipoprotein C3
ApoE: Apolipoprotein E
C-rp: C-reactive protein
DHA: Docosahexaenoic acid
DPA: Docosapentaenoic acid
EPA: Eicosapentaenoic acid
FA: Fatty acid
HbA1c: Glycated hemoglobin
HDL: High-density lipoprotein
HDL-c: High-density lipoprotein cholesterol
HMG-CoA: 3-hydroxy 3-methylglutaryl Coenzyme-A reductase
LC: Long-chain
LDL: Low-density lipoprotein
LDL-c: Low-density lipoprotein cholesterol
NHPs: Non-human primates
PCSK9: Proprotein convertase subtilisin/kexin type 9
VLDL: Very-low-density lipoprotein
